# Occurrence of randomly recombined functional 16S rRNA genes in *Thermus thermophilus* suggests genetic interoperability and promiscuity of bacterial 16S rRNAs

**DOI:** 10.1038/s41598-019-47807-z

**Published:** 2019-08-02

**Authors:** Kentaro Miyazaki, Natsuki Tomariguchi

**Affiliations:** 1 Department of Life Science and Biotechnology, Bioproduction Research Institute, National Institute of Advanced Industrial Science and Technology (AIST), Tsukuba, Ibaraki, 305-8566 Japan; 2 Department of Computational Biology and Medical Sciences, Graduate School of Frontier Sciences, The University of Tokyo, Kashiwa, Chiba, 277-8561 Japan; 3 Faculty of Life Sciences, Toyo University, Itakura, Gunma, 374-0193 Japan; 4 Present Address: Department of Computational Biology and Medical Sciences, Graduate School of Frontier Sciences, The University of Tokyo, Kashiwa, Chiba, 277-8561 Japan

**Keywords:** Evolution, Microbiology

## Abstract

Based on the structural complexity of ribosomes, 16S rRNA genes are considered species-specific and hence used for bacterial phylogenetic analysis. However, a growing number of reports suggest the occurrence of horizontal gene transfer, raising genealogical questions. Here we show the genetic interoperability and promiscuity of 16S rRNA in the ribosomes of an extremely thermophilic bacterium, *Thermus thermophilus*. The gene in this thermophile was systematically replaced with a diverse array of heterologous genes, resulting in the discovery of various genes that supported growth, some of which were from different phyla. Moreover, numerous functional chimeras were spontaneously generated. Remarkably, cold-adapted mutants were obtained carrying chimeric or full-length heterologous genes, indicating that horizontal gene transfer promoted adaptive evolution. The ribosome may well be understood as a patchworked supramolecule comprising patchworked components. We here propose the “random patch model” for ribosomal evolution.

## Introduction

**Figure 1:**
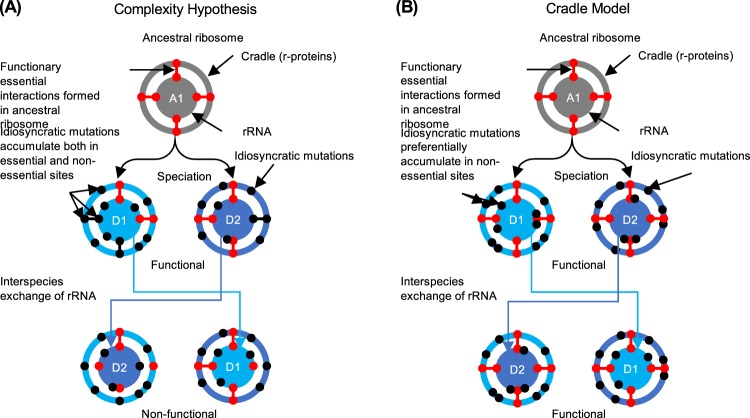
Evolutionary model for the bacterial ribosome. (**A**) The model supported by the complexity hypothesis. In this model, idiosyncratic mutations accumulate both in essential and non-essential sites of rRNA and cradle (r-proteins). Mutations occurring in functionally essential sites are quickly compensated by suppressive mutations; the ribosome retains function through co-evolution. Sequence specificity is ensured but rRNA are not interoperable between species because functionally essential interactions are species-specific and would be lost upon interspecies exchange. (**B**) The mechanism supported by the cradle model. In this model, during speciation, functionally essential interactions are locked-in and idiosyncratic mutations specifically accumulate at the non-essential sites; the ribosome retains function throughout evolution. Sequence specificity is ensured and rRNAs are interoperable between species because functionally essential interactions would be rewired upon interspecies exchange. A1, ancestral ribosome; D1, descendant ribosome-1; D2, descendant ribosome-2.

The translation system is one of the most ancient biological systems, traceable back to the prebiotic “RNA world”^1–6^. Among the components involved in translation, rRNAs, tRNAs, and large elongation factors were already in near-modern form in the “universal common ancestor” as evidenced by their omnipresence in all domains of life, i.e., bacteria, archaea, and eukaryotes^2^. By contrast, not all ribosomal proteins (r-proteins) were ubiquitous; the majority of them (15 in the small subunit and 19 in the large subunit) were universally distributed, but a minority were not^1,2,5,6^. This biased distribution shaped the specific architecture of ancestral ribosomes in each phylogenetic domain^2,6^. Surprisingly, despite billions of years of evolution from their ancestors, the general architecture of ribosomes (and other components involved in translation) remain largely unchanged within the domain; hybrid translation systems can be reconstituted using the components from well-evolved phylogenetically distinct species. More specifically, the translation factors of *E*. *coli* are capable of working cooperatively with ribosomes from *Bacillus subtilis*^7^ and *T*. *thermophilus*^8^. Thus, ribosomes have been shown to exhibit high functional modularity and interoperability in the bacterial translation system. However, it remains unknown whether ribosomal components, i.e., rRNAs and r-proteins, also have such high modularity and interoperability. In the past, these components—especially in 16S rRNA and 23S rRNA, localized at the centre of the ribosome—were not considered to harbour these characteristics due to their elaborate structural integrity within the supramolecule. According to the complexity hypothesis^9^, genes involved in complex biosystems, which are constrained by many interactions (as represented by ribosomes), tend to experience horizontal gene transfer (HGT) less frequently than those coding for products not involved in complex systems. Point mutations occurring in rRNAs during vertical inheritance are quickly fixed by suppressive or “compensatory neutral” mutations to retain function^10^. These idiosyncratic mutations gradually shaped the species-specificity of the gene, which became the basis of the use of 16S rRNA gene sequence for the phylogenetic classification of bacteria—indeed all living organisms on earth—based on the small subunit of rRNA^11,12^ (Fig. 1A).

Despite this consensus view, we recently showed that *E*. *coli* ribosomes accommodate heterologous 16S rRNAs^13–15^. More specifically, growth of the null mutant of *E*. *coli* was complemented by the 16S rRNA gene of Acidobacteria, which was different from Proteobacterial *E*. *coli* at the phylum level^15^. Further mutational analysis revealed that the vast majority of different nucleotides between Acidobacterial and *E*. *coli* 16S rRNAs were neutral to function, providing the first experimental evidence to support the neutral evolvability of rRNA genes^10^. Based on this interoperability of 16S rRNAs between *E*. *coli* and Acidobacteria, we proposed a new model for ribosomal evolution, designated the cradle model^15^ (Fig. 1B). In this model, functionally essential interactions between rRNA and its surrounding r-proteins or “cradle” were locked-in at an early stage of ribosomal evolution (at least prior to the branching point of Proteobacteria and Acidobacteria) and have remained unchanged thereafter^15^. Idiosyncratic mutations, including point mutations and insertions/deletions, occurred only in the non-essential sites, which rarely affected function. Accordingly, upon genetic exchange of 16S rRNA genes, essential interactions are rewired between the cradle and heterologous rRNA, resulting in the retention of function. The cradle model effectively explains the apparently opposing aspects of 16S rRNAs—sequence specificity and functional generality. However, the scope of the cradle model remains largely unknown. In this study, we attempted to verify the generality of the model in *T*. *thermophilus*^16,17^, an organism that is phylogenetically distantly related to *E*. *coli*. The availability of high-quality three-dimensional structures of ribosomes^18^ assists in the investigation.

## Results

### Genetic interoperability of 16S rRNA

Taking advantage of the high natural transformation (gene uptake and homologous recombination) efficiency of *T*. *thermophilus*^19^, we developed an experimental system to mimic HGT of 16S rRNA in the thermophile. First, a single knockout mutant was created to facilitate the functional characterization of heterologous 16S rRNAs. One of the two copies of the 16S rRNA gene (*rrsB*) in the genome was completely deleted to yield a mutant strain DB1 (Supplementary Fig. S1A). The remaining *rrsA* gene in the *T*. *thermophilus* DB1 genome was then targeted by heterologous 16S rRNA genes. The gene targeting vector (pUC4KrAHg1) carried a thermostable hygromycin B (Hg) resistant gene for the efficient selection of recombinants (Supplementary Fig. S1B). Several heterologous 16S rRNA genes were used as donors (see Table 1), including genes from the Deinococcus-Thermus phylum (*Deinococcus radiodurans*, *Meiothermus ruber*, and several *Thermus* species) as well as phylogenetically distinct moderately thermophilic bacteria, *Rhodothermus marinus* (Bacteroidetes) and *Rubrobacter xylanophilus* (Actinobacteria). We also used genes from the hyperthermophilic bacteria *Aquifex aeolicus* (Aquificae)^20^ and *Thermotoga maritima* (Thermotogae)^21^. Each of the 16S rRNA genes was PCR-amplified and replaced with the *rrsA* gene in pUC4KrAHg1. Experimental HGT was then performed by mixing each of the plasmids with *T*. *thermophilus* DB1 cells^19^; transformed cells were selected on LB/Hg agar plates. Several Hg-resistant transformants, designated DB2, were selected and the 16S rRNA genes therein were investigated.

**Table 1:** Source of 16S rRNA genes used in this study.

Sources	Phyla	% Identity to *T*. *thermophilus* 16S rRNA
*Thermus brockianus*	Deinococcus-Thermus	95.1
*Thermus kawarayensis*	Deinococcus-Thermus	96.6
*Thermus scotoductus*	Deinococcus-Thermus	93.8
*Thermus aquaticus*	Deinococcus-Thermus	96.3
*Meiothermus ruber*	Deinococcus-Thermus	85.9
*Deinococcus radiodurans*	Deinococcus-Thermus	80.4
*Rhodothermus marinus*	Bacteroidetes	78.5
*Rubrobacter xylanophilus*	Actinobacteria	80.7
*Aquifex aeolicus*	Aquificae	75.8
*Thermotoga maritima*	Thermotogae	79.3

**Figure 2:**
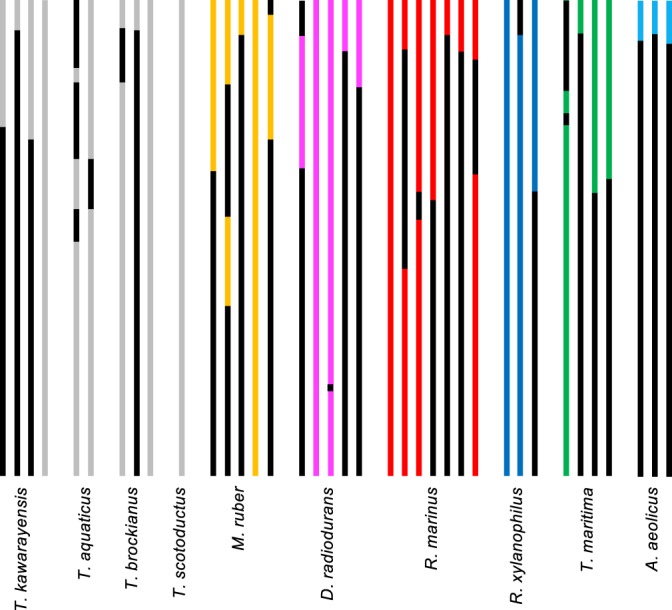
Schematic drawing of mutants. *T*. *thermophilus* sequence is coloured in black and donor sequences are in non-black. Clone names were omitted from the figure for simplicity; the order (from top to bottom for each donor) corresponds to that in Supplementary Table S1. Recombination boundaries scattered apparently at random over the entire sequence.

The entire list of the 16S rRNA genes included in the DB2 mutants is summarized in Supplementary Table S1, with schematic drawings provided in Fig. 2. Mutants with whole gene substitution were obtained for most of the donors, including those from different phyla, *R*. *marinus* and *R*. *xylanophilus*, but not for *A*. *aeolicus* and *T*. *maritima*. Reasons for the lack of full compatibility in the hyperthermophilic genes will be discussed later. *T*. *thermophilus*, *M*. *ruber*, and *D*. *radiodurans* belong to the same Deinococcus-Thermus phylum, but their optimal growth temperature ranges vary widely; extremely thermophilic *T*. *thermophilus* grows optimally at 70–75 °C, whereas moderately thermophilic *M*. *ruber* at 50–65 °C, and mesophilic *D*. *radiodurans* at 30 °C. Basically, different nucleotides are localized at the stem regions (Supplementary Fig. S2), wherein species-specificities are imprinted. The GC content of the different nucleotides was 75.9% for *T*. *thermophilus*, 51.9% for *M*. *ruber*, and 44.0% for *D*. *radiodurans*, respectively, whereas that of the consensus nucleotides was 59.5% (Supplementary Table S2); these tendencies were in agreement with the general rule of temperature adaptation of 16S rRNAs^22,23^. Despite the marked difference in the evolutionary pattern of each individual 16S rRNA, the “cold-adapted” *D*. *radiodurans* 16S rRNA was functional in the hybrid ribosome, suggesting that the surrounding *T*. *thermophilus* r-proteins (or “cradle”) congruently fit and reinforced the AU-rich RNA structure to protect the hybrid ribosome from thermal denaturation.

For *R*. *marinus* and *R*. *xylanophilus* 16S rRNAs, greater sequence diversity, including insertion/deletion, was observed particularly at helices h6, h9, h10, h17, and h26 (Supplementary Fig. S3); these helices are exposed to the molecular surface and have fewer interactions with r-proteins^18^. Accounting for the fully functional interoperability of *R*. *marinus* and *R*. *xylanophilus* 16S rRNAs in the *T*. *thermophilus* ribosome, these regions are functionally irrelevant as in the case of the *E*. *coli* ribosome^13^. *R*. *marinus* and *R*. *xylanophilus* should, despite the wide difference in organismal phylogeny, share essentially the same ribosomal architecture with *T*. *thermophilus*^18^.

However, hyperthermophilic 16S rRNAs were not functional in the *T*. *thermophilus* ribosome. Nevertheless, virtually all of the functionally essential nucleotides^24^ were conserved in the hyperthermophilic 16S rRNAs, except for two “mutations” in *T*. *maritima* 16S rRNA (G973C and C1210A, *E*. *coli* numbering). Accounting for the successful isolation of the chimeric mutants (S7 and S17 in Supplementary Table S1) carrying the two “mutations”, however, the effect of these “mutations” on the incomplete interoperability can be ruled out, though the exact reason remains obscure. Based on the genomic data, the *A*. *aeolicus* ribosome has an enlarged r-protein S19 (Supplementary Table S3). The protein consists of 219 amino acids—nearly double the size that of *T*. *thermophilus* S19 (93 amino acids). *A*. *aeolicus* S19 contains additional sequence at the N-terminus (128 amino acids) that did not match any known protein families (https://www.uniprot.org/uniprot/O66435). The calculated isoelectric point of the N-terminal sequence was 10.13, whereas that of the C-terminal sequence, which was homologous to other bacterial S19, was 10.40. Thus, the unique N-terminal region of *A*. *aeolicus* S19 likely interacts with some regions of rRNA in the native *A*. *aeolicus* ribosome. Total lengths of the 16S rRNA genes are also different: 1521 bases for *T*. *thermophilus* and 1587 bases for *A*. *aeolicus* (*cf*. 1560 bases for *T*. *maritima*). It is noteworthy that the genomes of *T*. *maritima* and *A*. *aeolicus* are the hybrid of archaea and bacteria^20,21^; the ribosomes may also have a hybrid nature. Taking the general architecture of archaeal ribosomes^25^ into account, additional archaeal-type RNA accretion and surface proteinization^3^ might have taken place in the hyperthermophilic ribosomes. Partially different architectures of hyperthermophilic ribosomes might explain the lack of full compatibility (and some successful chimerization) of the 16S rRNAs in *T*. *thermophilus* ribosome (Supplementary Fig. S2).

### Genetic promiscuity of 16S rRNA

**Figure 3:**
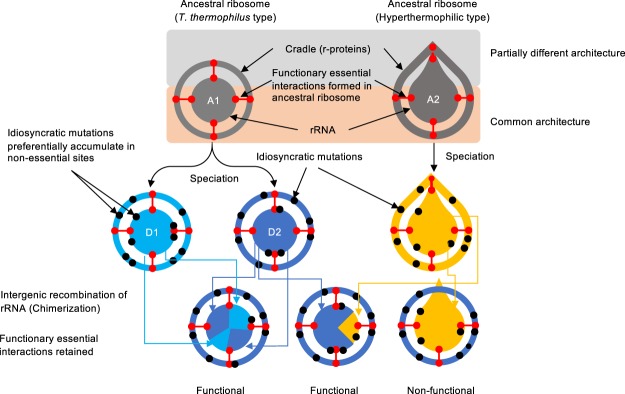
Random patch model for ribosomal evolution. We assume the presence of different types of ancestral bacterial ribosomes (A1 and A2). In the present study, there are two types: one is the *T*. *thermophilus* type (A1) and the other is the hyperthermophilic type (A2). Different architecture exists between the A1 and A2 ribosomes (shaded in grey). In the random patch model, chimerization is permitted between the 16S rRNAs (D1 and D2), which are cradled by the same architecture (A1 type). For ribosomes whose architecture is different (A2 type), the entire 16S rRNA is not transferrable but partial transfer would be permitted. A1, ancestral ribosome-1 (*T*. *thermophilus* type); A2, ancestral ribosome-2 (hyperthermophile type); D1, descendant ribosome-1; D2, descendant ribosome-2.

**Figure 4:**
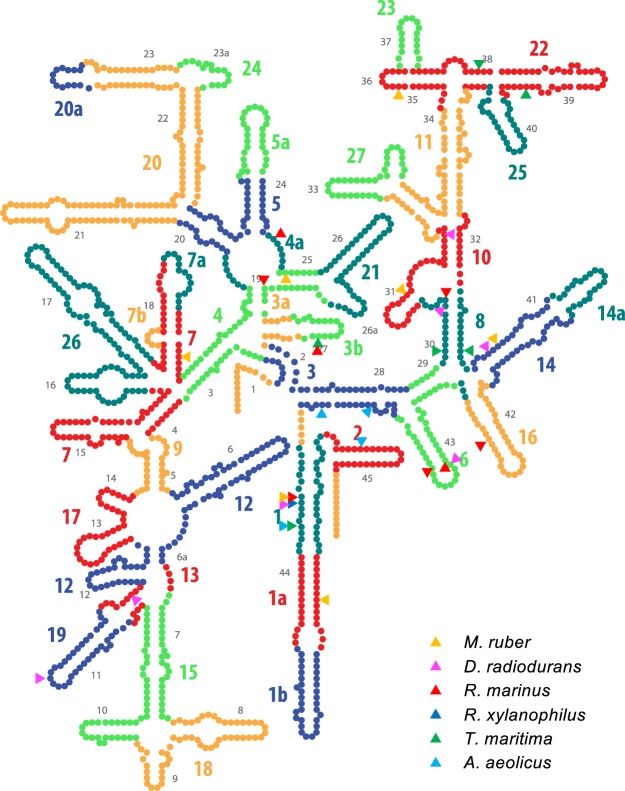
Boundary of chimeras mapped onto the secondary structure of 16S rRNA. Boundary (as defined by the first nucleotide that distinguishes two sequences) mapped onto the secondary structure of *T*. *thermophilus* 16S rRNA. In general, boundaries were scattered over the entire sequence (Fig. 2, Supplementary Table S1), but structurally tended to localize at the centre of the molecule, which was formed at the early stage of ribosomal evolution. Twenty-five out of a total of thirty-three boundaries were located at the 10 oldest segments.

As shown in Fig. 2 (and Supplementary Table S1), we identified chimeras that were spontaneously generated through homologous recombination in the thermophile. It is noteworthy that chimerization took place between the host *T*. *thermophilus* gene and all tested heterologous genes, including those from hyperthermophiles. *T*. *thermophilus* is a polyploid^26^, which is likely related to the high frequency of chimerization. Figure 3 illustrates the proposed mechanism for chimerization. Briefly, intergenic recombination (#1) followed by iterative cycles of intragenomic recombination (#2) between heterologous genomes effectively generated complex chimeras. In our experiments, allopolyploid (denoted as “Hetero” in Supplementary Fig. S4) status was often observed as evidenced by the appearance of mixed sequencing chromatogram, particularly when the sequence templates were prepared from fresh colonies (by colony PCR) (Supplementary Fig. S5). When the cells were grown to saturation and the sequence templates prepared from the purified genome, however, such mixed chromatograms disappeared, suggesting that the allopolyploid status would be genetically or phenotypically unfavourable. Local sequence identity required for chimerization was as short as 9 bases; such short stretches scatter over the entire sequence (see Supplementary Fig. S3 for example) (not limited to the present materials but also to any pairs of bacterial 16S rRNA genes), which would likely be the basis for the occurrence of random chimerization. For chimeric 16S rRNAs, recombination boundaries defined by the first discriminating nucleotide for two parents were mapped on the secondary structures of 16S rRNA as shown in Fig. 4 (original figure adopted from Supplementary Fig. S2 in the paper by Petrov *et al*.^3^) overlaid with the segment number proposed in the “accretion model^3^” which were sequentially ordered along the timeline of the formation of core structure in the primitive ribosome. According to the “accretion model”, the functional core was built by stepwise addition of ancestral expansion segments. In our chimeras, boundaries were scattered over the entire sequence (Fig. 2), but many of them were structurally localized in the ancestral expansion segments; 25 of 33 total boundaries were mapped on the first 10 “oldest” segments.

**Figure 5:**
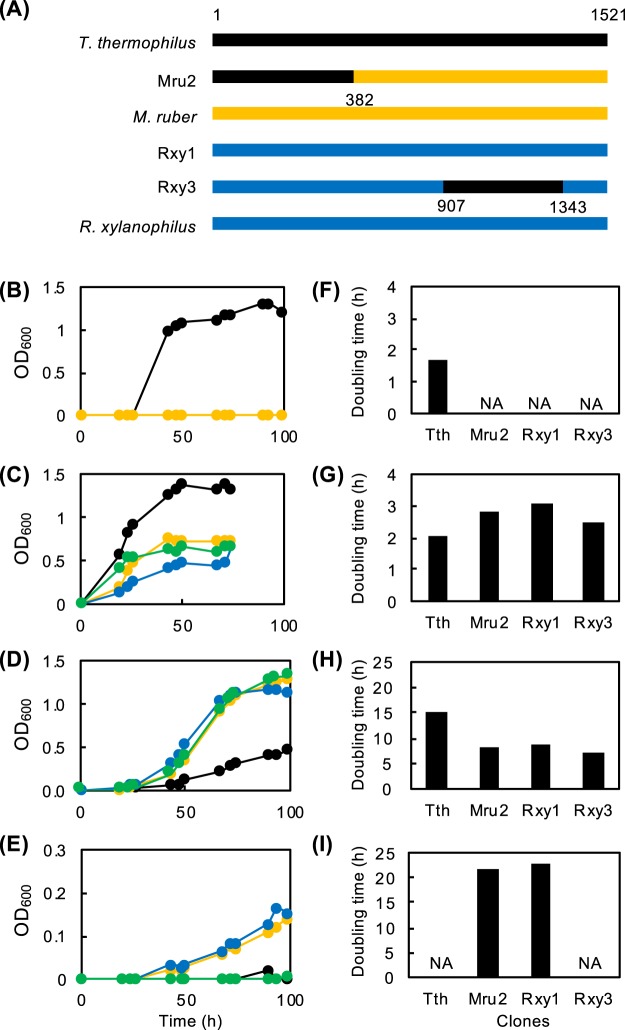
Cold-adapted mutant *T*. *thermophilus* DB2 strains. Fast growing mutants, DB2^Mru2^, DB2^Rxy1^, and DB2^Rxy3^ were obtained through enrichment cultivation at 50 °C. (**A**) Schematic drawing of the 16S rRNA genes of cold adapted mutants and parents. Numbers below the line indicate the boundary sites chimera. Rxy1 carries the full-length *R*. *xylanophilus* gene. Colours: *T*. *thermophilus* (black); *M*. *ruber* (yellow); *R*. *xylanophilus* (blue). (**B**–**D**) Growth curves of mutants and parental DB2 cells in LB/Hg at 75 °C (**B**) 70 °C (**C**), 50 °C (**D**) and 45 °C (**E**). (**F**–**I**) Doubling times (h) calculated from the growth curves (**B–D**) 75 °C (**F**) 70 °C (**G**) 50 °C (**H**) and 45 °C (**I**). Colours: DB2^Tth^ (black); DB2^Mru2^ (yellow); DB2^Rxy1^ (blue); DB2^Rxy3^ (green). NA, not available because no growth was observed.

One may think that such chimerization should cause large, if not lethal, fitness loss. To address this issue, we subjected each DB2 mutant library to growth competition. Experimentally, *T*. *thermophilus* DB1 cells were mixed with each plasmid for transformation; the bulk DB2 transformant library was directly inoculated in LB/Hg medium and grown at 50 °C, well below the optimal temperature for wildtype (70–75 °C)^16^. When the growth reached mid-log phase, cells were diluted (1/1000) in fresh LB/Hg and re-grown; this cultivation-dilution cycle was repeated five times to enrich cold-adaptive mutants (or fast growers). Cells were then singly isolated on LB/Hg agar plates and eight colonies were randomly selected from each library and sequenced. Strikingly, in the *M*. *ruber* library, all eight clones were chimeric with identical sequence (the representative clone is designated 16S^Mru2^ going forward); for *R*. *xylanophilus*, six of eight mutants contained the full-length sequence of the gene (16S^Rxy1^) and two contained identical chimeric sequence (16S^Rxy3^) (Fig. 5A). The growth of these three mutants DB2^Mru2^ (DB2 carrying 16S^Mru2^ noted as such going forward), DB2^Rxy1^, and DB2^Rxy3^ was characterized by the self-complemented DB2^Tth^ as a control. Growth curves are illustrated in Fig. 5B–E with the doubling times in Fig. 5F–I. As shown in Fig. 5B, all three mutants showed virtually no growth at 75 °C but DB2^Tth^ grew very well. At 70 °C, all mutants and DB2^Tth^ grew but the growth of DB2^Tth^ was far superior to the mutants (Fig. 5C). By contrast, at 50 °C, much faster growth was observed in the mutants relative to DB2^Tth^ (Fig. 5D). At 45 °C, growth was observed in the mutants but not in DB2^Tth^ (Fig. 5E), conclusively demonstrating the cold adaptation of the mutants. We searched the National Center for Biotechnology Information (NCBI) database using the 16S^Mru2^ sequence as a query and found a closely related sequence (FJ821631.1, uncultured bacterium clone K5), which was derived from a hot spring sediment. The 5′ (~210 b) and 3′ (~30 b) ends are missing in 16S^K5^ but the sequence (1262 bases) showed a striking overall similarity (99.4%) to 16S^Mru2^ with no large insertion/deletion (Fig. 5A).

## Discussion

According to Carl Woese, at the early stages of ribosome evolution, massive pervasive HGT dominated; aboriginal ribosomes were built up with loosely connected components^2,6^. Such “communal evolution”, however, ended relatively suddenly when the ribosome gained a refined function/structure through extensive trial and error. Once functionally refined ribosomes were built, the set of genes began to coevolve thereafter and function further improved^6^. Contrary to this evolutionary scenario for the ribosome, however, this study suggested the occurrence of HGT in 16S rRNAs. In our experimental mimic of evolutionary conditions, a diverse array of chimeric 16S rRNAs were generated nearly spontaneously between fully-refined extant ribosomes from phylogenetically distant species. Based on these results, we propose that communal evolution continued even after the establishment of refined structure/function of the ribosome. It is also noteworthy to point out that the outcome of HGT was not necessarily detrimental but in some cases functionally beneficial, as evidenced by the emergence of cold-adapted mutants: DB2^Mru2^, DB2^Rxy1^, and DB2^Rxy3^. It was particularly interesting for us to find that 16S^Mru2^, an artificial chimeric gene identified in the cold-adapted mutant, showed a striking sequence (99.4%) identity to the gene deposited in the database. We speculate that HGT-mediated chimerization is a valid, on-going strategy in nature.

Beyond our experimental approach, there are an increasing number of reports that support the occurrence of HGT in 16S rRNA genes in nature^27–29^. As shown in Supplementary Table S4, there seems to be no phylogenetic biases for the occurrence of HGT, which occurred in Proteobacteria, Firmicutes, Cyanobacteria, Actinobacteria, and Euryarchaeota of the domain Archaea, suggesting it is quite common in bacteria (and perhaps in archaea as well). There are two types of HGT: one is the coexistence of heterogeneous 16S rRNA genes (with sequences typically different by more than 2%) in a single genome and the other is the presence of chimeric 16S rRNA genes, which may have originated following the mechanism described in this paper, i.e., intergenomic recombination followed by iterative intragenomic recombination (Supplementary Fig. S4). All these instances were discovered by careful inspection of the gene sequences by researchers (and thus were extremely labour intensive and low throughput, accordingly), but many more instances are likely to have eluded researchers’ manual screening. The major obstacle limiting the identification of such recombination-driven gene evolution (beyond the prejudice that such events are unlikely to occur) is largely determined by the analytical method. Current methods employed for phylogenetic analysis (e.g., neighbour-joining, maximum likelihood, and maximum parsimony) are based on point mutation-driven tree-shape evolution as a primary model, but it is intrinsically inappropriate to decipher the evolutionary history for genes driven by recombination. By using an appropriate approach such as phylogenetic network analysis^30^, it would be possible to untangle complex evolutionary history including HGT. In fact, through phylogenetic network analysis^30^, we revealed the frequent occurrence of 16S rRNA HGT in the genus *Enterobacter*^31^. Briefly, out of ten *Enterobacter* species (whose genomic sequences were completely analysed), we found three ancestral 16S rRNA groups; the others were likely created through recursive recombination between the ancestors and chimeric descendants. We also found the occurrence of HGT between different genus. *Yersinia enterocolitica* (NC 015224.1; genomic locus, 4158244–4159734) contains a chimeric sequence with the 3′-half transferred from *E*. *coli* (NC 000913.2; genomic locus, 4206170–4207711). Likewise, *Citrobacter koseri* (NC_009792.1; genomic locus, 4325568-4327114) carries a chimeric 16S rRNA genes between *E*. *coli* (NC_009801.1; genomic locus, 4360914–4362454) and *Salmonella enterica* (NC_011094.1; genomic locus, 3976169–3977702). Ironically, extensive HGT in rRNA genes would mask the recombination signal (a sequence segment carrying consecutive nucleotide variants introduced by other species through HGT), making it difficult to distinguish recombination signal from point mutations introduced during vertical inheritance. The ever-expanding 16S rRNA gene database would also cause the same outcome; increasing numbers of sequence entries prohibit distinguishing the recombination signal from point mutations in pairwise comparisons, clouding the evolutionary mechanism.

In addition to rRNAs, there are several reports that suggest the occurrence of HGT in r-proteins^32–35^. The r-proteins that are essential to function (i.e., evolutionary conservative and lacking in species-specificity), should be interoperable in a given ribosome (e.g., L12^36^, S18^36^, and L2^37^); such r-proteins would be amenable to HGT. Those that are not essential to function (thus optional, hence deletable^38^) would also be amenable to HGT. In fact, Chen *et al*. reported that r-protein S4, one of the universal r-proteins^5^ essential for the initiation of small subunit ribosomal assembly and translational accuracy, seems to have evolved through HGT^34^. Likewise, a universal protein S14^35^ and non-essential protein L27^32^ also seem to have undergone HGT. Interestingly, all of these instances involve HGT of the entire r-protein molecules; to the best of our knowledge, there are no reports documenting the chimerization of r-proteins. Unlike base pair-dominated RNAs, proteins are assembled with a variety of interactions (salt-bridge, hydrophobic interaction, aromatic ring stacking, electrostatic interaction, disulphide bridge, etc.), the complexities of which must have worked as a functional barrier to chimerization (fully supported by the complexity hypothesis^9^). If not totally impossible, very careful guidance is required to design functional pieces (structurally compact and continuous in sequence) and to reconstitute them into a whole protein^39^, which is highly unlikely to take place as the result of a natural random process, particularly in r-proteins.

Though it is certainly true that HGT introduces large genetic perturbations, this does not necessarily mean it immediately introduces large functional perturbations; if the molecules share common architectures as in the case of rRNAs, recombination should be even less harmful than point mutations. Promiscuity and interoperability, both of which were simultaneously observed in this study, should be based on the same principle—the common architecture of bacterial ribosomes. Such robust architecture might have been selected through massive pervasive HGT from the earliest stage of ribosomal evolution. 16S rRNAs may thus be considered dynamically evolving, highly promiscuous molecules driven by HGT. The ribosome may well be understood as a randomly patchworked supramolecule assembled with randomly patchworked components. We here propose the “random patch model” of ribosomal evolution as an advanced version of the cradle model^15^, promoting the Copernican Revolution with our vision of the evolutionary mechanism of rRNA and the ribosome.

## Materials and Methods

### Reagents

KOD FX Neo DNA polymerase was purchased from Toyobo (Osaka, Japan). EmeraldAmp PCR Master Mix and In-Fusion^®^ Cloning Kit were purchased from Takara Bio (Kusatsu, Japan). Restriction enzymes and DNA modification enzymes were purchased from New England Biolabs (Ipswich, MA, USA). Oligonucleotides were purchased from Thermo Fisher Scientific (Waltham, MA, USA). 5-Fluoroorotate (5-FOA) was purchased from Sigma-Aldrich (St. Louis, MO, USA). Agar, ampicillin (Amp), and hygromycin B (Hg) were purchased from Wako Pure Chemical/FUJIFILM (Osaka, Japan). Lennox LB [1% (w/v) tryptone, 0.5% (w/v) yeast extract_,_ 1% (w/v) NaCl] was purchased from Nacalai (Kyoto, Japan).

### Bacterial strains and growth conditions

*E*. *coli* JM109 competent cells were purchased from RBC Bioscience (Taiwan, China). *T*. *thermophilus* HB27 was a gift from M. Tamakoshi. *R*. *marinus* and *R*. *xylanophilus* were isolated from the Arima hot spring in Kobe, Japan by us (Tomariguchi and Miyazaki, unpublished results). Agar was used to solidify the medium at 1.6% (w/v). Hg was added at 50 µg/ml and Amp was added at 100 µg/ml when necessary.

### Knockout of *ttc3024* (*rrsB*) gene

*T*. *thermophilus* strain HB27 carries two identical 16S rRNA genes located at 1 765 087–1,766,607 for *rrsA* and 1 310 193–1 311 713 for *rrsB* in the genome, respectively^40^. Unlike the case in most bacteria, the three rRNA genes (i.e., 16S, 23S, and 5S rRNAs) do not constitute an operon in *T*. *thermophilus* HB27^41^; both 16S rRNA genes are separated from the 23S-5S gene clusters in the genome. A single knockout mutant of *T*. *thermophilus* HB27 was created by deleting *rrsB* from the genome (Supplementary Fig. S1A). To amplify the *rrsB* gene with its flanking regions, a set of primers TTC1383Rev and TTC1380Rev were used (primer sequences provided in Table S5). The PCR mixture contained 25 µl of 2 × PCR buffer, 10 µl of 4 mM dNTPs, 10 µl of water, 1.5 µM of each primer, 10 ng of genomic DNA, and 1 U of KOD FX Neo DNA polymerase in 50 µl. The sample was heated at 94 °C for 2 min, followed by 30 cycles of incubation at 94 °C for 10 s, 68 °C for 2.5 min, and a final incubation at 68 °C for 5 min. The fragment (*ca*. 4.3 kb) was agarose gel-purified then cloned into the SmaI site of pUC18. The resultant plasmid, which carried the *rrsB* gene in the opposite direction of the lac promoter of pUC18 was named pUC4KrB1. Based on this vector, a deletion construct was generated. It is noteworthy that *ttc1381* codes orotidine-5′-monophosphate decarboxylase (gene name, *pyrF*), which is involved in pyrimidine biosynthesis. Genetic inactivation of the gene is well known to confer resistance to the pyrimidine analogue 5-FOA in many bacteria including *T*. *thermophilus*^42^. Therefore, we deleted the entire *rrsB* sequence together with the partial sequence (100 bp each from the 5′ end) of the flanking genes through inverse PCR by using primer set TTC1381_100OUT and TTC1382_100OUT (Supplementary Fig. S1A) with the following temperature cycles: 94 °C for 2 min followed by 30 cycles of incubation at 94 °C for 10 s, 68 °C for 4 min, and a final incubation at 68 °C for 5 min. The reaction mixture was treated with DpnI (10 U), agarose gel-purified, and circularized in the presence of T4 DNA kinase and T4 DNA ligase at room temperature. A portion of the mixture was introduced into competent *E*. *coli* JM109 cells and grown on LB/Amp agar plates at 37 °C. Several colonies were sequenced to obtain an integration vector pUC4KrBΔrrsB1O1O which was then used to transform *T*. *thermophilus* HB27, following the procedure described previously^19^. Mutant strains lacking the entire *rrsB* gene were selected on minimum medium plates containing 200 µg/ml 5-FOA at 70 °C. Twelve 5-FOA-resistant colonies were picked, the genomic DNA was purified from each strain, and DNA sequence surrounding the deletion site was confirmed. The knockout mutant thus obtained was named *T*. *thermophilus* DB1 (*∆rrsB*, *pyrF’*, 5-FOA^R^).

### Gene targeting for *rrsA*

The *rrsA* gene located at 1 765 087–1 766 607 in the genome was replaced by heterologous 16S rRNA genes by homologous recombination. To amplify the *rrsA* gene with its flanking regions, a set of primers, TTC1858Fwd and TTC1861Fwd, were used. The fragment (*ca*. 4.2 kb) was then cloned into the SmaI site of pUC18. The resultant plasmid, designated pUC4KrA1, was then inverse-PCR amplified using a set of primers TTC1859UpRev and TTC1859Fwd300, resulting in the deletion of a portion of the TTC1859 gene (nucleotide positions 1 to 300). The fragment was treated with DpnI to eliminate the templated DNA and 5′-dephosphorylated with alkaline phosphatase. Next, a gene cassette of a thermostable Hg resistant gene (Hg^R^) was amplified using a set of primers HgFwd and HgRev. The amplicon was gel-purified, 5′-phosphorylated y using T4 DNA kinase, then ligated with the linearized pUC4KrA1vector in the presence of T4 DNA ligase. A desired recombinant plasmid was selected on LB/Amp/Hg agar plates at 37 °C. The resultant plasmid was named pUC4KrA1Hg and used for subsequent gene displacement experiments.

Heterologous 16S rRNA gene was PCR-amplified from various bacterial genomes as listed in Table 1. A set of primers, Thermus_1F and Thermus_1521R, was used to amplify the gene (except for the hyperthermophiles) using KOD FX neo DNA polymerase. For the genes from hyperthermophiles, a set of primers Thermus_1F(AT) and Thermus_1499R(AT) was used. PCR cycling conditions were as follows: 94 °C for 2 min, followed by 30 cycles of incubation at 94 °C for 10 s, 68 °C for 1 min, and a final incubation at 68 °C for 5 min. Amplicons were gel-purified and dissolved in 30 µl of water. For the vector, pUC4KrAHg1 was PCR-amplified using a set of primers Thermus_1R and Thermus_1521F (except for the hyperthermophiles) following the temperature cycles: 94 °C for 2 min, followed by 30 cycles of incubation at 94 °C for 10 s, 68 °C for 4 min, and a final incubation at 68 °C for 5 min. For hyperthermophiles, a set of primers Thermus_1R(AT) and Thermus_1499F(AT) was used. The reaction mixture was treated with DpnI (10 U) at 37 °C overnight and the products were agarose gel-purified and dissolved in 30 µl of water. The vector (1 µl) and insert (1 µl) fragments were mixed and ligated using the In-Fusion Cloning Kit to yield a final volume of 10 µl. The reaction mixture (1 µl) was then introduced into competent *E*. *coli* JM109 cells and grown on LB/Amp agar plates at 37 °C. Several colonies were picked and the plasmid was purified from the cells, then the correct construction of the vector was confirmed by DNA sequencing.

Using the heterologous 16S rRNA genes, gene knockout experiments were carried out using the *T*. *thermophilus* DB1 as a host. In theory, depending on the recombination sites, various genes would be obtained (Supplementary Fig. S1B). Mutants carrying the whole heterologous 16S rRNA gene would be obtained when recombination takes place at Region-1 and -4, whereas chimeras would be obtained when recombination takes place at Region-2 and -4. When recombination takes place at Region-3 and -4, the gene sequence would remain intact. Transformation was made by mixing *T*. *thermophilus* DB1 cells with pUC4KrAHg1 (carrying the heterologous 16S rRNA genes). After incubation at 60 °C for 2 h, cells were spread on LB/Hg agar plates and incubated at 50–70 °C. After 24–48 h, several single colonies were picked and grown in LB/Hg broth, then genomic DNA was purified using the QIAamp genomic DNA Kit (Qiagen, Venlo, Netherlands) following the manufacturer’s instructions. In certain cases, to quickly check the sequence of 16 S rRNA genes, we conducted colony-PCR. The PCR mixture contained EmeraldAmp PCR Master Mix, 2.5 µM each of TTC1859UpRev and TTC1860DwnFwd primers, and a tiny amount of colony as a template. The cycling conditions were: 98 °C for 2 min followed by 35 cycles of incubation at 98 °C for 10 s and 68 °C for 2 min. Amplicons were gel-purified and used as templates for DNA sequencing. DNA sequence analysis was performed using the Sanger method on an Applied Biosystems automatic DNA sequencer (ABI PRISM 3130xl Genetic Analyzer) with an Applied Biosystems BigDye (ver. 3.1) Kit (Applied Biosystems/Thermo Fisher Scientific. Foster City, CA, USA).

### Growth competition

For growth competition experiments, DB1 cells (100 µl) were transformed by mixing them with each of the plasmids and cultivating at 50 °C for 2 h in LB. Cells were then inoculated in fresh LB/Hg (5 ml). After growing the cells to mid-log phase at 50 °C, they were diluted in fresh LB/Hg (5 ml) and re-grown. This sub-cultivation process was repeated 5 times then cells were singly isolated on LB/Hg plates at 50 °C. For each donor, eight colonies were randomly picked and subjected to DNA sequencing analysis. Growth curves were generated by growing the cells at 50 °C in LB/Hg; OD_600_ was monitored at certain intervals.

### Sequence analysis

A web-based BLAST search^43^ was carried out using the National Center for Biotechnology Information (NCBI/NIH, Bethesda, MD, USA) nucleotide database nucleotide collection (nr/nt), with the program selection optimized for highly similar sequences (megablast)^44^.

## Supplementary information

Supporting Information

## Data Availability

DNA sequence data reported in this study have been deposited in the DNA Data Bank of Japan (DDBJ) database, http://www.ddbj.nig.ac.jp (accession nos. LC436693 – LC436740, LC440032, LC440033, and LC440343).
