# Supplementary material for: Occurrence of randomly recombined functional 16S rRNA genes in Thermus thermophilus suggests genetic interoperability and promiscuity of bacterial 16S rRNAs
Source: Sci Rep. 2019 Aug 2;9:11233. doi: 10.1038/s41598-019-47807-z (PMC6677816; doi:10.1038/s41598-019-47807-z)
Supplement: Supplementary file 1 — Supporting Information [file 41598_2019_47807_MOESM1_ESM.pdf]

Supplementary Information

Occurrence of randomly recombined functional 16S rRNA genes in *Thermus thermophilus* suggests genetic interoperability and promiscuity of bacterial 16S rRNAs

Kentaro Miyazaki and Natsuki Tomariguchi

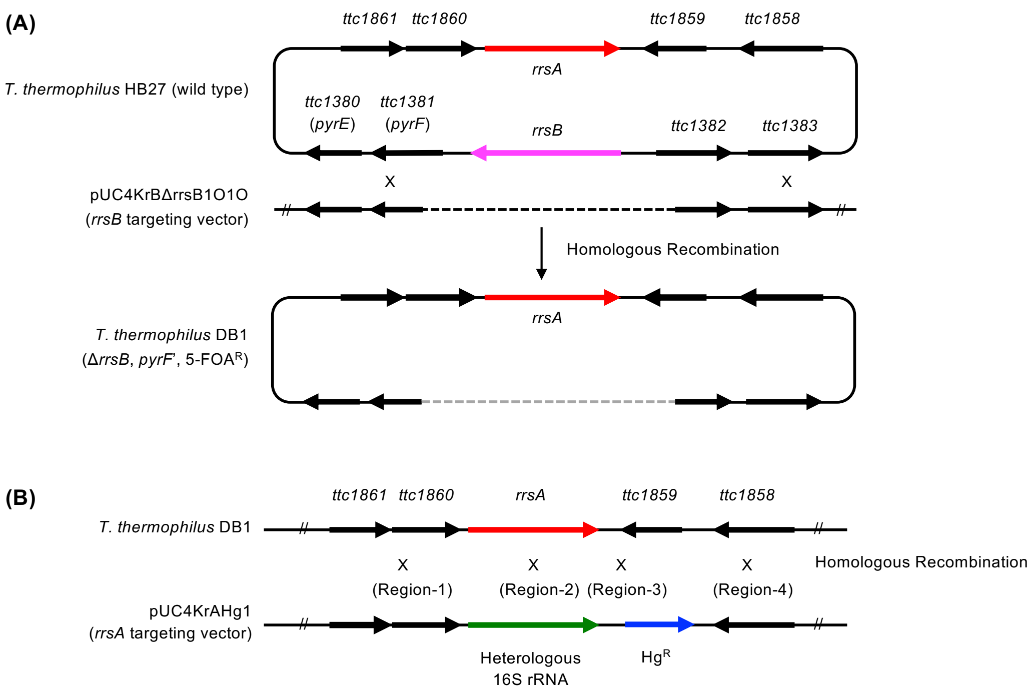

Supplementary Fig. S1. Experimental horizontal gene transfer of 16S rRNA in *T. thermophilus*. (**A**) Creation of 16S rRNA gene knockout mutant of *T. thermophilus* HB27. A targeting vector (pUC4KrBΔrrsB1O1O) was used to transform *T. thermophilus* HB27. Through a double crossover mechanism, a mutant strain designated DB1 was obtained. Given that the flanking *ttc1381* codes *pyrF*, by partially deleting the gene, the *rrsB*-deletion mutant was obtained as a 5-FOA resistant. (**B**) Interspecies exchange of 16S rRNA gene. *T. thermophilus rrsA* gene was replaced by a heterologous gene. *ttc1859*, which is nonessential for growth, was replaced by a thermostable Hg^R gene for efficient screening of recombinants.

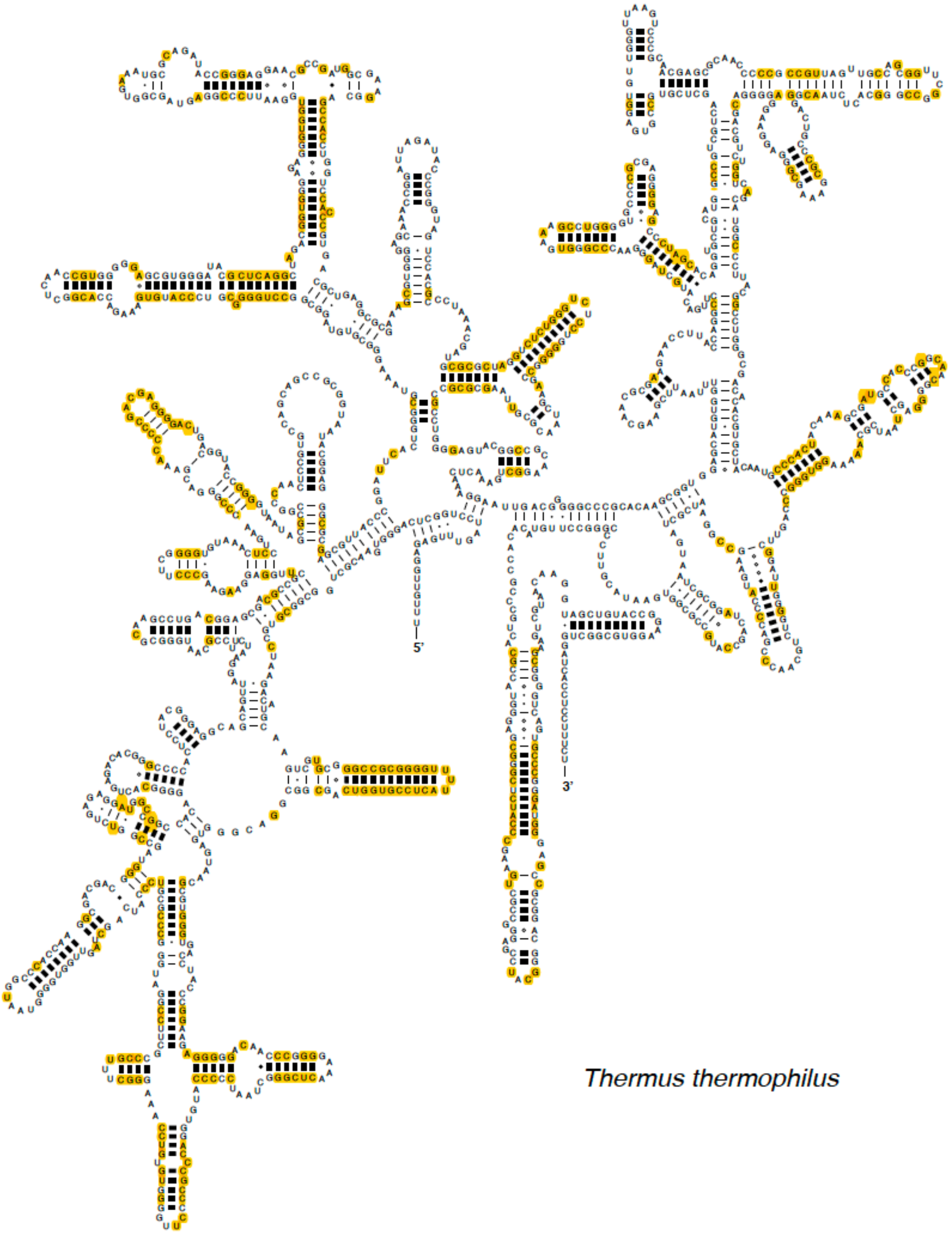

Supplementary Fig. S2. Different nucleotides in *M. ruber* and *D. radiodurans* 16S rRNAs mapped on the secondary structure of *T. thermophilus* 16S rRNA. Variable sites are shown in yellow. Large portions of the different nucleotides localized at the stem regions.

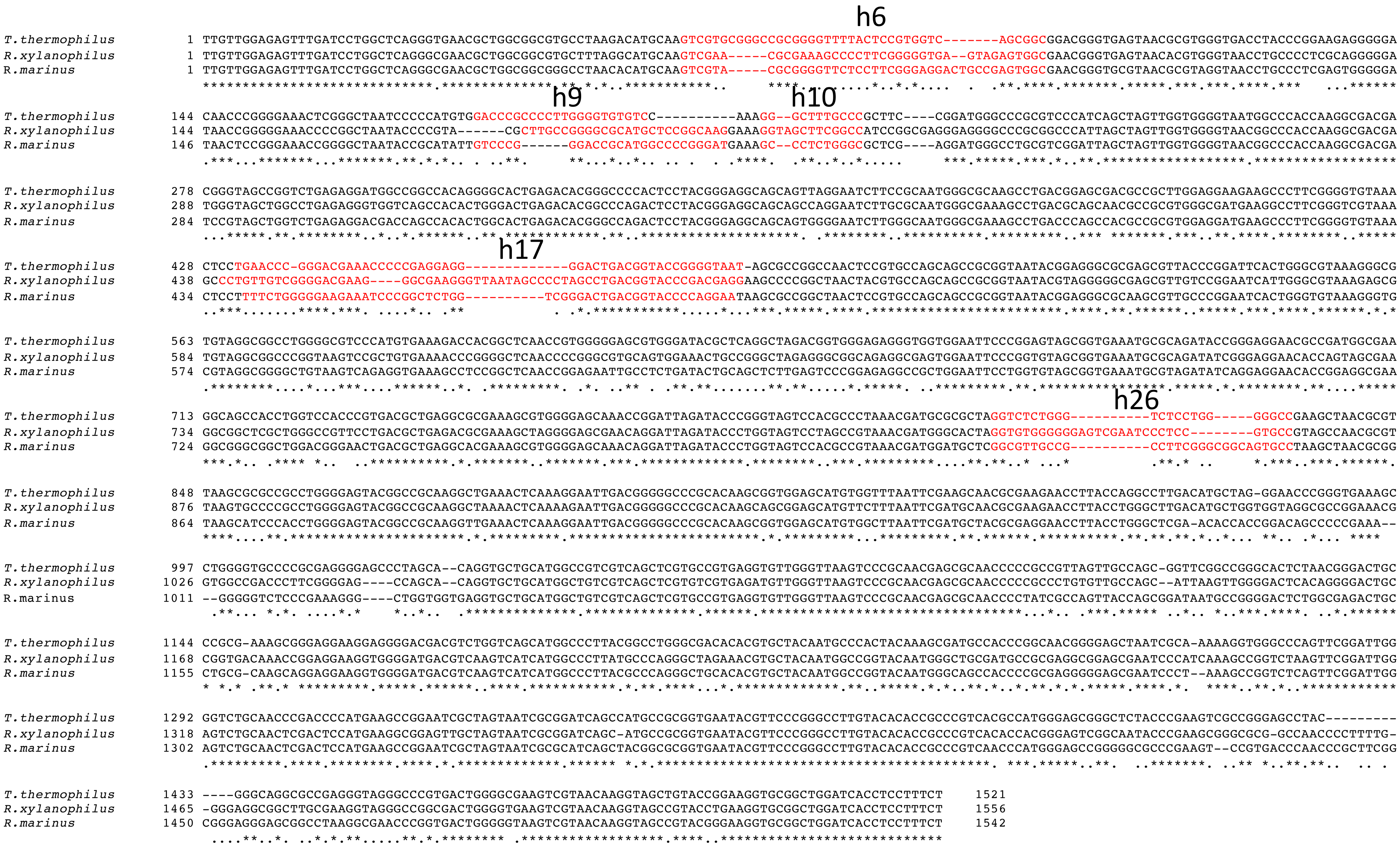

Supplementary Fig. S3. Multiple sequence alignment of *T. thermophilus*, *R. marinus*, and *R. xylanophilus* 16S rRNAs. Highly variable helices with insertion/deletions are coloured in red. Helices containing insertion/deletions were labeled.

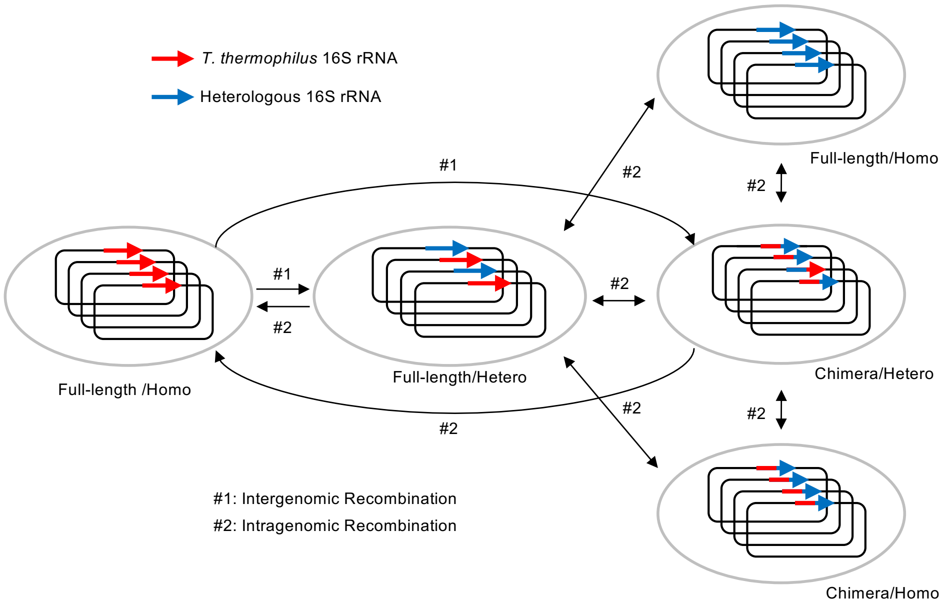

Supplementary Fig. S4. Mechanism for generation of chimera in *T. thermophilus.* *T. thermophilus* is a polyploid. Intergenomic followed by iterative intragenomic recombination promotes chimerization of 16S rRNA genes.

```
T.thermophilus AGACATGCAAGTCGTGCGGGCCGCGGGGTTTTACTCCGTGGTCAGCGGCGGACGGGTGAGTAACGCGTGG 120
D.radiodurans  AGACATGCAAGTCGAACGCGGT-------CTT----CGGACCGAGTGGCGCACGGGTGAGTAACACGTAA 109
               **********  ** *          **     *     ** **** ************* ***
```

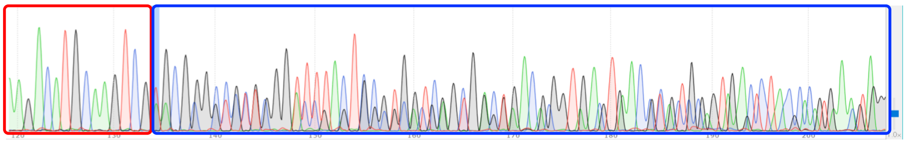

Supplementary Fig. S5. An example of mixed sequencing chromatograms. *T. thermophilus* is a polyploid and in some cases, allopolyploid status was observed as a mixed sequencing chromatogram in a heterogeneous region. In this figure, *T. thermophilus* and *D. radiodurans* sequences share identical sites but the different sites show superimposed sequences that may reflect the presence of insertions/deletions.

Supplementary Table S1. List of *T. thermophilus* DB2 mutants

Donors<br>Mutants	Chimera (Type) [1] or Full-length	Boundaries [2]	Database ID
T. kawarayensis			LC436704
TY2	Chimera (HD)	1115	LC436735
TY5	Chimera (HD)	1424	LC436727
TY15	Chimera (HD)	1075	LC436739
TY16	Full-length	N.A.[3]	LC436729
T. aquaticus			LC440032
TY10	Chimera (DHDHDH)	748/846/1006/1251/1297	LC436726
TY23	Chimera (DHD)	846/1006	LC436717
T. brockianus			LC436697
TY1	Chimera (DHDHD)	262/265/1251/1424	LC436725
TY11	Chimera (HD)	1424	LC436732
TY12	Full-length	N.A.[3]	LC436719
T. scotoductus			LC436703
TY3	Full-length	N.A.[3]	LC436721
M. ruber			LC436693
TY9	Chimera (HD)	974	LC436709
TY19	Chimera (HDHD)	543/822/1244	LC436695
TY20	Chimera (HD)	1409	LC436700
TY25	Full-length	N.A.[3]	LC436711
TY40	Chimera (HDH)	1075/1474	LC436714
Mru2	Chimera (HD)	382	LC440033
D. radiodurans			LC436701
TY32	Chimera (DHD)	989/1410	LC436728
TY34	Full-length	N.A.[3]	LC436723
TY38	Chimera (HDH)	264/286	LC436715
S52	Chimera (HD)	1357	LC436736
S66	Chimera (HD)	1242	LC436699
R. marinus			LC436722
42	Full-length	N.A.[3]	LC436737
48	Chimera (DHD)	955/1356	LC436702
53	Chimera (DHD)	812/904	LC436696
S40	Chimera (HD)	881	LC436731
S41	Chimera (HD)	1409	LC436734
S44	Chimera (HD)	1356	LC436740

S86	Chimera (DHD)	955/1326	LC436738
R. xylanophilus			LC436710
61	Full-length	N.A.[3]	LC436724
63	Chimera (DH)	1410	LC436720
89	Chimera (HD)	906	LC436712
Rxy3	Chimera (DHD)	907/1343	LC440343
T. maritima			LC436716
S1	Chimera (HDHD)	1116/1152/1232	LC436698
S2	Chimera (HD)	1414	LC436713
S7	Chimera (HD)	904	LC436694
S17	Chimera (HD)	949	LC436707
A. aeolicus			LC436706
S4	Chimera (HD)	1393	LC436705
S8	Chimera (HD)	1414	LC436730
S10	Chimera (HD)	1383	LC436718
S29	Chimera (HD)	1510	LC436708

[1] Donor sequence is denoted by D and host *T. thermophilus* sequence is denoted by H.

[2] Boundary is defined by the first nucleotide site that distinguishes two sequences. For example, the TY2 chimeric mutant from 5' end to nucleotide position 1114 derives from *T. thermophilus* (H) and from 1115 to 3' end from *T. kawarayensis* (D). *E. coli* numbering.

[3] N.A., not applicable.

Supplementary Table S2. Nucleotide composition of *T. thermophilus*, *M. ruber*, and *D. radiodurans* 16S rRNA genes

		A	T	G	C	%GC	Total
Full-length	*T. thermophilus*	311	237	546	427	0.64	1521
	M. ruber	347	288	488	368	0.574	1491
	D. radiodurans	367	304	483	350	0.554	1504
Consensus		260	188	391	267	0.595	1106
Variable	*T. thermophilus*	51	49	155	160	0.759	415
	M. ruber	87	100	97	101	0.514	385
	D. radiodurans	107	116	92	83	0.44	398

Supplementary Table S3. Molecular mass of ribosomal proteins in 30S subunit [1]

Proteins	*T. thermophilus*	*A. aeolicus*	*T. maritima*
S1	59851.6	60769.2	61356.7
S2	29228.3	31429.0	29887.9
S3	26672.6	24137.2	24057.8
S4	24306.0	24796.6	24702.3
S5	17525.2	23192.7	18461.4
S6	11954.6	13455.3	15351.3
S7	17997.7	18606.5	13439.3
S8	15791.3	19439.6	15270.7
S9	14369.4	17046.5	15179.4
S10	11911.8	12217.2	11618.6
S11	13694.6	13508.5	14074.2
S12	15119.7	14188.4	14042.4
S13	14312.6	14321.6	14413.6
S14	7121.6	7485.0	7324.9
S15	10536.2	10888.7	10789.7
S16	10766.2	13020.2	11235.9
S17	12278.4	12817.8	12549.4
S18	10241.1	8694.2	8997.9
S19	10563.3	21624.1	10880.8
S20	11684.9	13249.3	11274.1
Total	345927.0	374887.5	344908.4

[1] Genomic sequences used for calculation were as follows: AE017221.1 (*T. thermophilus*); NC_000918.1 (*A. aeolicus*); NC_000853.1 (*T. maritima*).

Supplementary Table S4. Natural occurrence of HGT in 16S rRNAs

Phylogeny	Type of HGT	References
Haloarcula marismortui (Euryarchaeota*)*	Intragenomic heterogeneity	Mylvaganam and Dennis, 1992 [45]
Aeromonas (Proteobacteria)	Chimeragenesis	Sneath et al., 1993 [46]
Rhizobium (Proteobacteria)<br>*Aeromonas* (Proteobacteria)	Chimeragenesis	Eardly et al., 1996 [47]
Escherichia and *Salmonella* (Proteobacteria)	Intragenomic heterogeneity<br>Chimeragenesis	Cilia et al., 1996 [48]
Thermobispora bispora (Actinobacteria)	Intragenomic heterogeneity	Wang et al., 1997 [49]
Vibrio cholerae (Proteobacteria)	Intragenomic heterogeneity	Lan and Reeves, 1998 [50]
Streptomyces (Actinobacteria)	Chimeragenesis	Ueda et al., 1999 [51]
Thermomonospora chromogena (Actinobacteria)	Intragenomic heterogeneity<br>Chimeragenesis	Yap et al., 1999 [52]
Actinomycetes (Actinobacteria)	Chimeragenesis	Wang and Zhang, 2000 [53]
Bradyrhizobium (Proteobacteria)	Chimeragenesis	Parker, 2001 [54]
Streptococcus anginosus (Firmicutes)	Chimeragenesis	Schouls et al., 2003 [55]
Rhizobia (Proteobacteria)	Chimeragenesis	van Berkum et al. [56]
Thermoanaerobacter tengcongensis (Firmicutes)	Intragenomic heterogeneity	Acinas et al., 2004 [57]
Desulfotomaculum kuznestovii (Firmicutes)	Intragenomic heterogeneity	Tuorova, et al., 2001 [58]
Halosimplex carlsbadense (Euryarchaeota)<br>*Natrinema* sp. (Euryarchaeota*)*	Intragenomic heterogeneity	Boucher et al., 2004 [59]

Rhizobium galegae (Proteobacteria)	Chimeragenesis	Eardly et al., 2005 [60]
Helicobacter (Proteobacteria)	Chimeragenesis	Dewhirst et al., 2005 [61]
Scytonema hyalinum (Cyanobacteria)	Intragenomic heterogeneity	Johansen et al., 2017 [62]

Supplementary Table S5. Oligonucleotide primers used in this study

Oligonucleotides	Sequence (5' to 3')
TTC1380Rev	CTAGACCTCCTCCAAGGGCACC
TTC1383Rev	CTACACGTCTAGGATGACCTGGGC
TTC1381_100OUT	ACACCCTCGAGCTCCTCGAGGCCCTGGCCC
TTC1382_100OUT	TCACCCTGACCCGGGGGGAAGCCGGGAGAA
TTC1858Fwd	ATGAGCATCCCCGAGGAGCTTTGGCGCCGC
TTC1860DownFwd	TAGCATGGCTTTTGCTGCGCGGGCG
TTC1861Fwd	ATGTCACGGGACCTTCTGGGCCTCGAGGGG
TTC1859UpRev	ACCCCCTCCTTACGGCAACAAAAAA
TTC1859Fwd300	CTCATGCGCCACCTGGGGGG
Thermus_1F	TTGTTGGAGAGTTTGATCCTGGCTCAGGG
Thermus_1521R	AGAAAGGAGGTGATCCAGCCGCACC
Thermus_1R	CCCTGAGCCAGGATCAAACTCTCCAACAA
Thermus_1521F	GGTGCGGCTGGATCACCTCCTTTCT
Thermus_1F(AT)	TTTGTTGGAGAGTTTGATCCTGGCT
Thermus_1499R(AT)	GGAGGTGATCCAGCCGCA
Thermus_1R(AT)	AGCCAGGATCAAACTCTCCAACAAA
Thermus_1499F(AT)	TGCGGCTGGATCACCTCC
HgFwd	ATGAAAAAGCCTGAACTCACCGTG
HgRev	TTAGGCTGCCAGGTCGACGGAT

References

45 Mylvaganam, S. & Dennis, P. P. Sequence heterogeneity between the two genes encoding 16S rRNA from the halophilic archaebacterium *Haloarcula marismortui*. *Genetics* **130**, 399-410 (1992).

46 Sneath, P. H. Evidence from *Aeromonas* for genetic crossing-over in ribosomal sequences. *Int J Syst Bacteriol* **43**, 626-629, doi:10.1099/00207713-43-3-626 (1993).

47 Eardly, B., Wang, F. & Berkum, P. v. Corresponding 16S rRNA gene segments in Rhizobiaceae and *Aeromonas* yield discordant phylogenies. *Plant Soil* **186**, 69-74, doi:10.1007/978-94-011-5700-1_9 (1996).

48 Cilia, V., Lafay, B. & Christen, R. Sequence heterogeneities among 16S ribosomal RNA sequences, and their effect on phylogenetic analyses at the species level. *Mol Biol Evol* **13**, 451-461, doi:10.1093/oxfordjournals.molbev.a025606 (1996).

49 Wang, Y., Zhang, Z. & Ramanan, N. The actinomycete *Thermobispora bispora* contains two distinct types of transcriptionally active 16S rRNA genes. *J Bacteriol* **179**, 3270-3276 (1997).

50 Lan, R. & Reeves, P. R. Recombination between rRNA operons created most of the ribotype variation observed in the seventh pandemic clone of *Vibrio cholerae*. *Microbiology* **144**, 1213-1221, doi:10.1099/00221287-144-5-1213 (1998).

51 Ueda, K., Seki, T., Kudo, T., Yoshida, T. & Kataoka, M. Two distinct mechanisms cause heterogeneity of 16S rRNA. *J Bacteriol* **181**, 78-82 (1999).

52 Yap, W. H., Zhang, Z. & Wang, Y. Distinct types of rRNA operons exist in the genome of the actinomycete *Thermomonospora chromogena* and evidence for horizontal transfer of an entire rRNA operon. *J Bacteriol* **181**, 5201-5209 (1999).

53 Wang, Y. & Zhang, Z. Comparative sequence analyses reveal frequent occurrence of short segments containing an abnormally high number of non-random base variations in bacterial rRNA genes. *Microbiology* **146**, 2845-2854, doi:10.1099/00221287-146-11-2845 (2000).

54 Parker, M. A. Case of localized recombination in 23S rRNA genes from divergent bradyrhizobium lineages associated with neotropical legumes. *Appl Environ Microbiol* **67**, 2076-2082, doi:10.1128/aem.67.5.2076-2082.2001 (2001).

55 Schouls, L. M., Schot, C. S. & Jacobs, J. A. Horizontal transfer of segments of the 16S rRNA genes between species of the *Streptococcus anginosus* group. *J Bacteriol* **185**, 7241-7246, doi:10.1128/jb.185.24.7241-7246.2003 (2003).

56 van Berkum, P. *et al.* Discordant phylogenies within the rrn loci of Rhizobia. *J Bacteriol* **185**, 2988-2998 (2003).

57 Acinas, S. G., Marcelino, L. A., Klepac-Ceraj, V. & Polz, M. F. Divergence and redundancy of 16S rRNA sequences in genomes with multiple rrn operons. *J Bacteriol* **186**, 2629-2635, doi:10.1128/JB.186.9.2629-2635.2004 (2004).

58 Turova, T. P., Kuznetsov, B. B., Novikova, E. V., Poltaraus, A. B. & Nazina, T. N. Heterogeneity of nucleotide sequences of 16S ribosomal RNA genes from the *Desulfotomaculum kuznetsovii* type strain. *Mikrobiologiia* **70**, 788-795 (2001).

59 Boucher, Y., Douady, C. J., Sharma, A. K., Kamekura, M. & Doolittle, W. F. Intragenomic heterogeneity and intergenomic recombination among haloarchaeal rRNA genes. *J Bacteriol* **186**, 3980-3990, doi:10.1128/jb.186.12.3980-3990.2004 (2004).

60 Eardly, B. D., Nour, S. M., van Berkum, P. & Selander, R. K. Rhizobial 16S rRNA and dnaK genes: mosaicism and the uncertain phylogenetic placement of *Rhizobium galegae*. *Appl Environ Microbiol* **71**, 1328-1335, doi:10.1128/aem.71.3.1328-1335.2005 (2005).

61 Dewhirst, F. E. *et al.* Discordant 16S and 23S rRNA gene phylogenies for the genus *Helicobacter*: implications for phylogenetic inference and systematics. *J Bacteriol* **187**, 6106-6118, doi:10.1128/jb.187.17.6106-6118.2005 (2005).

62 Johansen, J. R. *et al.* Highly divergent 16S rRNA sequences in ribosomal operons of *Scytonema hyalinum* (Cyanobacteria). *PLoS One* **12**, e0186393, doi:10.1371/journal.pone.0186393 (2017).
